# Production and control of coagulation proteins for factor X activation in human endothelial cells and fibroblasts

**DOI:** 10.1038/s41598-020-59058-4

**Published:** 2020-02-06

**Authors:** Clay T. Cohen, Nancy A. Turner, Joel L. Moake

**Affiliations:** 1 Department of Pediatrics, Section of Hematology-Oncology, Baylor College of Medicine, Texas Children’s Hospital, Houston, TX USA; 2 Department of Bioengineering, Rice University, Houston, TX USA

**Keywords:** Membrane proteins, Mechanisms of disease

## Abstract

Human endothelial cells (ECs) synthesize, store, and secrete von Willebrand factor multimeric strings and coagulation factor (F) VIII. It is not currently known if ECs produce other coagulation factors for active participation in coagulation. We found that 3 different types of human ECs in primary culture produce clotting factors necessary for FX activation via the intrinsic (FVIII-FIX) and extrinsic (tissue factor [TF]-FVII) coagulation pathways, as well as prothrombin. Human dermal fibroblasts were used as comparator cells. TF, FVII, FIX, FX, and prothrombin were detected in ECs, and TF, FVII, FIX, and FX were detected in fibroblasts. In addition, FVII, FIX, FX, and prothrombin were detected by fluorescent microscopy in EC cytoplasm (associated with endoplasmic reticulum and Golgi proteins). FX activation occurred on human umbilical vein EC surfaces without the addition of external coagulation proteins, proteolytic enzymes, or phospholipids. Tumour necrosis factor, which suppresses the generation of activated protein C and increases TF, augmented FX activation. Fibroblasts also produced TF, but (in contrast to ECs) were incapable of activating FX without the exogenous addition of FX and had a marked increase in FX activation following the addition of both FX and FVII. We conclude that human ECs produce their own coagulation factors that can activate cell surface FX without the addition of exogenous proteins or phospholipids.

## Introduction

Human endothelial cells (ECs) help maintain blood flow and prevent extra-vascular blood loss. The precise molecular contributions of ECs to haemostasis are unknown. We conducted experiments to determine if human ECs can produce coagulation proteins. Comparative experiments were performed with human fibroblasts, sub-EC vascular wall components that are exposed to blood upon vascular wall injury.

For many years hepatocytes were considered the predominant (or exclusive) site of coagulation factor production^1–9^. In recent murine and human studies, however, liver sinusoidal ECs (LSECs), rather than hepatocytes, were demonstrated to be the primary cellular source of factor (F) VIII^10–13^. Other extra-hepatic vascular ECs, including human umbilical vein ECs (HUVECs) and human glomerular microvascular ECs (GMVECs), have also been shown to produce FVIII^14–18^. FVIII is stored in EC Weibel-Palade bodies (WPBs), and secreted along with ultra-large (UL) von Willebrand factor (VWF) multimers^18^. The FVIII data suggest that ECs may have a more active role in coagulation factor production than previously appreciated.

ECs produce surface regulatory proteins that prevent excessive coagulation. These include thrombomodulin (TM), endothelial protein C receptor (EPCR), tissue factor pathway inhibitor (TFPI), and protein C (PC)^19–22^. TM-bound thrombin converts PC that is bound to EPCR into activated protein C (APC). APC (and protein S [PS]) inactivates activated FVIII and FV^23–25^ and, therefore, limits the functions of FVIII-FIX (intrinsic tenase complex) and FX-FV (prothrombinase complexes). TFPI inhibits the tissue factor (TF)-FVII (extrinsic tenase complex) activation of FX^26–28^.

Previous *in vitro* studies in the presence of human external purified or plasma coagulation proteins showed that EC and fibroblast surfaces contribute to the activation of FX and fibrin clot formation^29–32^. Additional investigation showed that EC surfaces contain FIX binding sites^33–35^ and are capable of inflammatory cytokine-induced TF expression^36,37^. In contrast to ECs, TF is constitutively expressed on fibroblast cell surfaces^38,39^. Fibroblasts do not, however, produce either FVIII or VWF^18^. There are no previous reports that human ECs produce coagulation proteins and activate coagulation reactions on their surfaces without the addition of external coagulation proteins.

## Results

We first compared coagulation protein production, in cell lysates and released into supernatants, from three types of human ECs with data obtained similarly from human fibroblasts.

### Quantification of TF, FVII, FIX, FX, and prothrombin in untreated EC and fibroblast lysates

**Figure 1:**
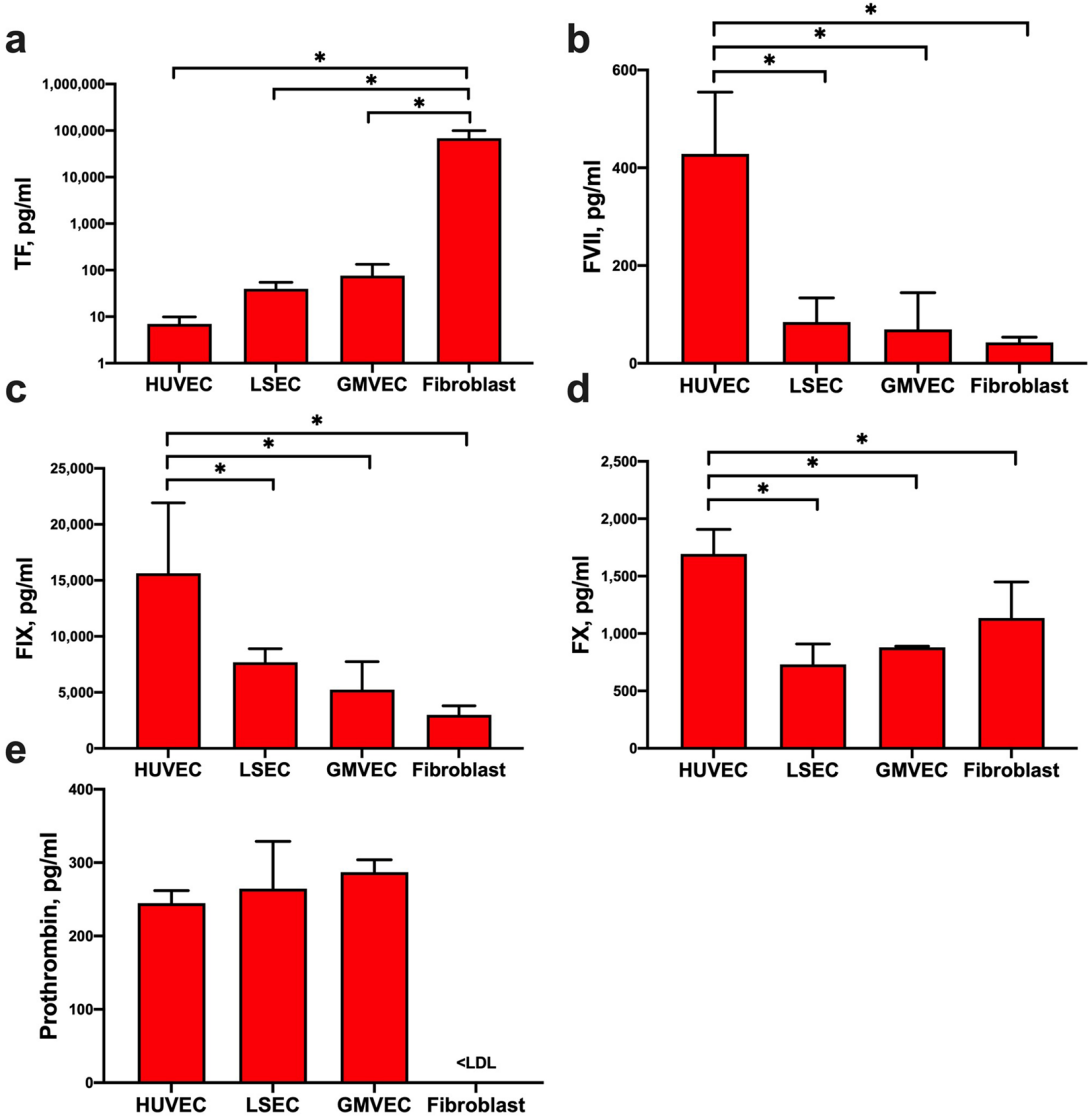
Comparison of TF, FVII, FIX, FX, and prothrombin protein levels measured in untreated ECs and fibroblasts. Cell lysates for quantification of (**a**) TF, (**b**) FVII, (**c**) FIX, (**d**) FX, and (**e**) prothrombin by ELISA were prepared from HUVECs (n = 3–8), LSECs (n = 3–4), GMVECs (n = 3–4), and fibroblasts (n = 3–5). Measured protein concentrations (means + SD) in pg/ml were normalized to total lysate protein to account for cell number difference. Values below the lowest detectable limit of the assay are noted as <LDL. *p < 0.05.

The protein levels of TF, FVII, FIX, FX, and prothrombin were measured in the lysates of untreated GMVECs, HUVECs, LSECs, and fibroblasts using commercial immunoassays. The immunoassay antibodies detect only human proteins (and not bovine coagulation factors). The measured protein values were normalized to total protein in cell lysates to account for cell number differences. Fibroblasts produced 900-fold more TF than GMVECs (p = 0.0001), 1,700-fold more than LSECs (p = 0.0001), and 9,700-fold more than HUVECs (p < 0.0001, Fig. 1a). HUVECs produced 5-fold more FVII than LSECs (p = 0.0004), 6-fold more than GMVECs (p < 0.0001), and 10-fold more than fibroblasts (p < 0.0001, Fig. 1b). HUVECs also produced 2–3-fold higher amounts of FIX than LSECs (p = 0.049) and GMVECs (p = 0.0087), and 5-fold more FIX than fibroblasts (p = 0.0041, Fig. 1c). Additionally, HUVEC FX levels were 2-fold higher than both GMVECs (p = 0.0021) and LSECs (p = 0.0003), and 1.5-fold higher than fibroblasts (p = 0.024, Fig. 1d). Levels of prothrombin were similar in HUVEC, LSEC, and GMVEC lysates (Fig. 1e). In contrast, prothrombin was undetectable in fibroblast lysates.

We have previously demonstrated in both HUVECs and GMVECs the presence of intracellular FVIII and the secretion of FVIII in complex with ULVWF^18^. We did not study the production of the contact system of coagulation proteins, as their deficiencies have not been associated with bleeding^40–42^.

In summary, human ECs (HUVECs, GMVECs, and LSECs) contain FVII, FIX, FX, TF, and prothrombin. Human fibroblasts have FVII, FIX, and FX, although prothrombin is absent. Fibroblasts also produce TF concentrations that are many-fold greater than in ECs.

### Quantification of FVII, FIX, FX, and prothrombin released into supernatants of untreated ECs and fibroblasts

**Figure 2:**
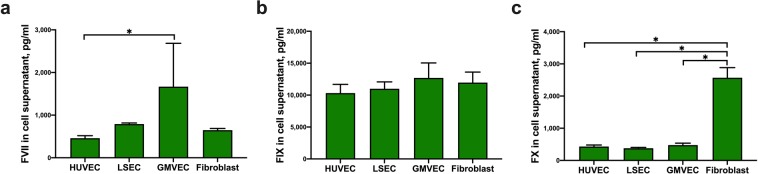
Quantification of FVII, FIX, and FX released into the supernatants of untreated ECs and fibroblasts. HUVEC, LSEC, GMVEC, and fibroblast supernatants were collected from cells incubated for 24 hours in serum-free media and quantified by ELISA; (**a**) FVII, (**b**) FIX, and (**c**) FX. Graphs show protein concentrations in pg/ml (means + SD) for HUVECs (n = 4), GMVECs (n = 3–4), LSECs (n = 3–4), and fibroblasts (n = 3). *p < 0.05.

Concentrations of coagulation proteins FVII, FIX, FX, and prothrombin were measured in supernatants of untreated HUVECs, LSECs, GMVECs, and fibroblasts collected from 24-hour serum-free media using commercial immunoassays. GMVEC supernatant contained 3.5-fold more FVII than HUVEC supernatant (p = 0.04, Fig. 2a). Measured levels of FIX were similar in EC and fibroblast supernatants (Fig. 2b). Levels of FX in fibroblast supernatant were 5–7-fold higher than FX in supernatants of the ECs under study (p < 0.0001, Fig. 2c). Prothrombin levels were below detection in each EC supernatant tested. Prothrombin was not measured in fibroblast supernatants because the protein was undetectable in fibroblast lysates.

In summary, human FIX, FX, and FVII were detected in EC and fibroblast supernatants, whereas released prothrombin was undetectable in each cell supernatant tested.

Further analysis of EC coagulation proteins required the use of verified monospecific antibodies for coagulation protein immunofluorescent microscopy detection and co-localization studies with ER and Golgi markers to corroborate intracellular production.

### Confirmation of coagulation antibodies specificity

The specificity of the polyclonal sheep antibodies made against purified human FVII, FIX, and FX and subsequently used for microscopic detection, was confirmed by Western blotting. The blots showed that each primary antibody detected only the specified standard protein target (Supplementary Fig. S1). The purpose of the Western blots was to show that the sheep polyclonal antibodies against human coagulation proteins were specific and did not detect any other proteins made in the ECs. We loaded samples of each purified coagulation protein and GMVEC lysates into different gel lanes. Antibodies against the human coagulation proteins detected only the appropriate standard and did not detect any protein in the EC lysate. We concluded that the coagulation proteins were not made in sufficient quantities to allow detection by these antibodies. Our results also indicate that the coagulation proteins antibodies did not cross-react with proteins produced in ECs at high concentrations (e.g., VWF).

We also verified that the sheep polyclonal antibodies made against human coagulation factors FIX and FX used for the immunofluorescence microscope studies do not detect bovine coagulation factors FIX and FX (Supplementary Fig. S2). Human FIX protein and bovine FIX protein, immobilized in 96-well plates with concentrations ranging from 15–1,000 ng/ml, were detected using polyclonal sheep antibody against human FIX, followed by secondary donkey anti-sheep-HRP and a fluorescent HRP substrate. Each concentration of the human FIX was detected, and the fluorescent signal increased linearly with concentration. In contrast, the immobilized bovine FIX detection was non-linear, and was only detected at the highest concentrations, (with ~8-fold lower intensities than human FIX). The polyclonal sheep antibody against human FX was also tested for detection of the same concentration range of immobilized human FX and bovine FX. Similar results were obtained showing that this sheep anti-human FX antibody does not detect bovine FX (Supplementary Fig. S2).

In summary, the Western blot data demonstrates that the polyclonal sheep antibodies against human coagulation factors FVII, FIX, and FX are specific and do not detect any other EC produced protein. In addition, the sheep antibodies against human FIX and FX do not detect bovine FIX or bovine FX.

### Coagulation proteins prothrombin, FVII, FIX, and FX are detectable within LSECs, GMVECs, and HUVECs

**Figure 3:**
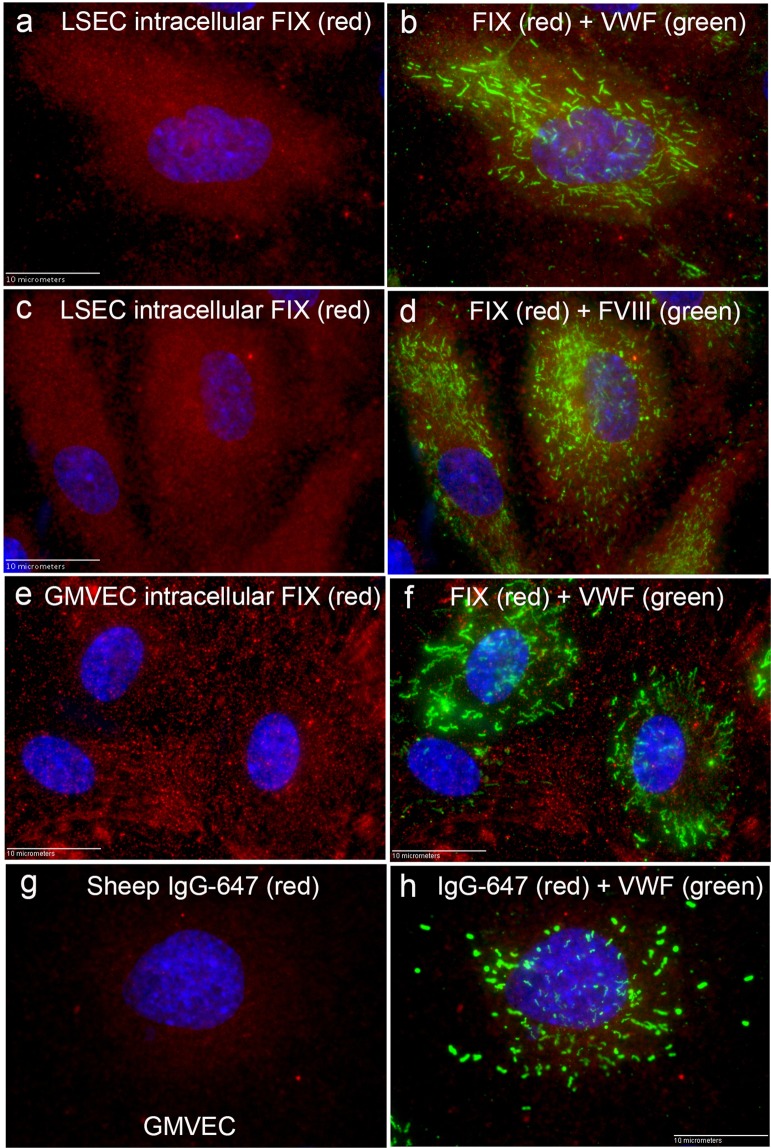
Fluorescent detection of FIX plus VWF, or FIX plus FVIII in GMVECs and LSECs. Confluent LSECs and GMVECs on coverslips were fixed and treated with Triton-X to allow intracellular staining. Cells in (**a–f**) were first stained with polyclonal sheep antibody to human FIX plus secondary goat anti-sheep IgG Alexa Fluor (AF)-647 (red). Washed cells were subsequently stained to detect VWF or FVIII. (**a**) LSECs stained for FIX, and in (**b**) The merged image after staining for VWF in WPBs with rabbit anti-human VWF plus chicken anti-rabbit IgG AF-488 (green). (**c**) LSECs stained for FIX, and in (**d**) the merged image after FVIII detection in WPBs using mouse anti-human FVIII + goat anti-mouse IgG AF-488 (green). (**e**) GMVECs stained for FIX, and in (**f**) the merged image after staining for VWF. In (**g**) GMVECs were stained with secondary goat anti-sheep IgG AF-647 (red) alone, and in (**h**) the merged image after VWF detection. GMVECs and LSECs were imaged at 100×, nuclei were detected with DAPI (blue), and are representative of 8–9 images. Calibration bar is 10 microns.

Coagulation factors prothrombin, FVII, FIX, and FX were detected by fluorescent microscopy in the cytoplasm of LSECs, GMVECs, and HUVECs using verified human-specific polyclonal sheep antibodies plus secondary fluorescent-conjugated antibodies. Figure 3 shows fluorescent detection of intracellular FIX and VWF in WPBs (to confirm cell type) in LSECs and GMVECs, as well as FIX and FVIII detection in LSECs. GMVECs stained with secondary detection antibodies alone show only background fluorescence (Fig. 3g). The merged image of background fluorescence plus VWF detection is shown in Fig. 3h. The fluorochromes on the secondary detection antibodies (Alexa Fluor [AF]-647 and AF-488) used in these studies have widely separated wavelengths for excitation and emission. We have previously demonstrated the absence of fluorescent “cross-talk” or “bleed-through” between these channels in our microscope system^18,43^.

**Figure 4:**
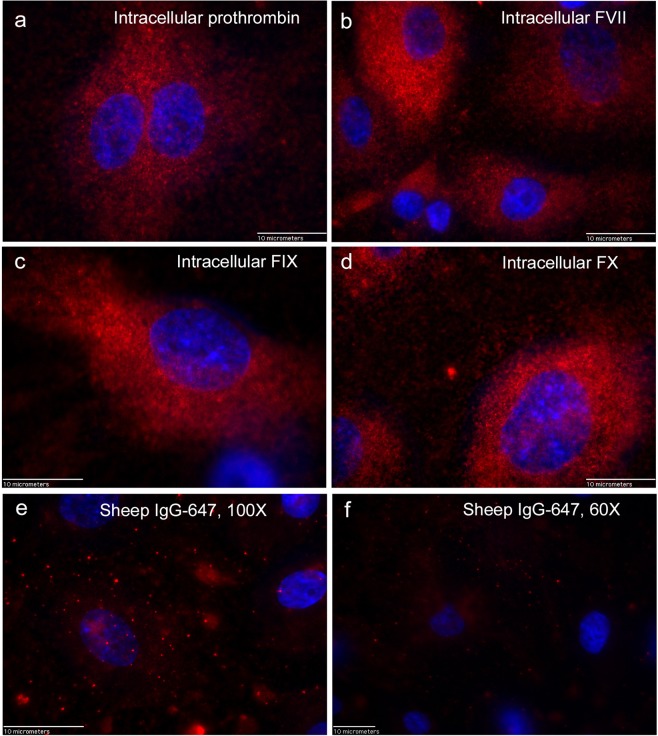
Fluorescent detection of intracellular prothrombin, FVII, FIX, and FX in HUVECs. Confluent HUVECs on coverslips were fixed and treated with Triton-X to allow intracellular staining. Cells were stained with polyclonal sheep antibodies either to human: (**a**) prothrombin, (**b**) FVII, (**c**) FIX, or (**d**) FX plus secondary goat anti-sheep IgG AF-647 (red). HUVECs were imaged at 100× and nuclei were detected with DAPI (blue). HUVECs in (**e)** and (**f**) were stained with the secondary goat anti-sheep IgG AF-647 (red) alone plus DAPI at 100× (**e**) and 60× (**f**). Calibration bar is 10 microns.

Additional images demonstrating intracellular detection of prothrombin, FVII, FIX, and FX in HUVECs plus other secondary detection controls are shown in Fig. 4.

In summary, using fluorescent microscopy and monospecific antibodies to prothrombin, FIX, FX, and FVII, indicates the presence of these coagulation proteins in human ECs.

### Intracellular prothrombin, FVII, FIX, and FX proteins co-localize with endoplasmic reticulum (ER) and Golgi proteins in ECs

Using immunofluorescent microscopy and non-overlapping secondary antibody pairs, coagulation factors prothrombin, FVII, FIX, and FX were detected throughout the EC cytoplasm and were usually associated with both the ER and the Golgi (Table 1). The cellular association was determined by calculations of Pearson’s (PCC) and Manders’ (M1 and M2) colocalization correlation coefficients in merged images of each coagulation factor with the ER or Golgi. The PCC measurement describes the number of locations within the merged image with similar red and green intensity levels, while M1 and M2 express the fraction of each channel probe that overlaps with the other channel probe, independent of signal intensity. Table 1 shows positive co-localization results in HUVECs of the coagulation factors with protein disulphide-isomerase (PDI, known ER protein), as well as positive colocalization with Golgi proteins.

**Table 1:** Positive co-localization of prothrombin, FVII, FIX, and FX with Golgi and ER proteins in HUVECs.

	Prothrombin and Golgi, mean ± SD (range)	Prothrombin and PDI, mean ± SD (range)
PCC	0.63 ± 0.09 (0.47–0.78)	0.48 ± 0.22 (0.14–0.82)
M1	0.72 ± 0.17 (0.46–0.98)	0.75 ± 0.09 (0.64–0.89)
M2	0.81 ± 0.09 (0.66–0.98)	0.83 ± 0.07 (0.74–0.94)
# of cells analyzed	55	139
	**FVII and Golgi, mean ± SD (range)**	**FVII and PDI, mean ± SD (range)**
PCC	0.52 ± 0.26 (0.12–0.83)	0.40 ± 0.20 (0.12–0.73)
M1	0.83 ± 0.09 (0.55–0.93)	0.73 ± 0.16 (0.47–0.92)
M2	0.90 ± 0.04 (0.76–0.95)	0.82 ± 0.17 (0.52–0.98)
# of cells analyzed	145	208
	**FIX and Golgi, mean ± SD (range)**	**FIX and PDI, mean ± SD (range)**
PCC	0.56 ± 0.19 (0.23–0.85)	0.70 ± 0.07 (0.55–0.85)
M1	0.79 ± 0.07 (0.63–0.89)	0.69 ± 0.08 (0.50–0.80)
M2	0.82 ± 0.08 (0.66–0.92)	0.69 ± 0.08 (0.49–0.81)
# of cells analyzed	148	130
	**FX and Golgi, mean ± SD (range)**	**FX and PDI, mean ± SD (range)**
PCC	0.73 ± 0.09 (0.53–0.83)	0.39 ± 0.23 (0.10–0.77)
M1	0.85 ± 0.08 (0.70–0.96)	0.80 ± 0.14 (0.55–0.95)
M2	0.88 ± 0.08 (0.73–0.99)	0.87 ± 0.12 (0.63–0.98)
# of cells analyzed	68	136

Colocalization coefficients, Pearson’s (PCC) and Manders’ (M1 and M2), were measured in HUVEC images stained concurrently with antibodies against Golgi and PDI (protein disulphide-isomerase) proteins and antibodies detecting coagulation factors prothrombin, FVII, FIX, and FX. The entire microscope image at 60× was used to analyze the extent of co-localization between each coagulation factor with Golgi and PDI proteins using the antibody pairs detailed in the Fluorescence colocalization measurement section within the Materials and Methods.

**Figure 5:**
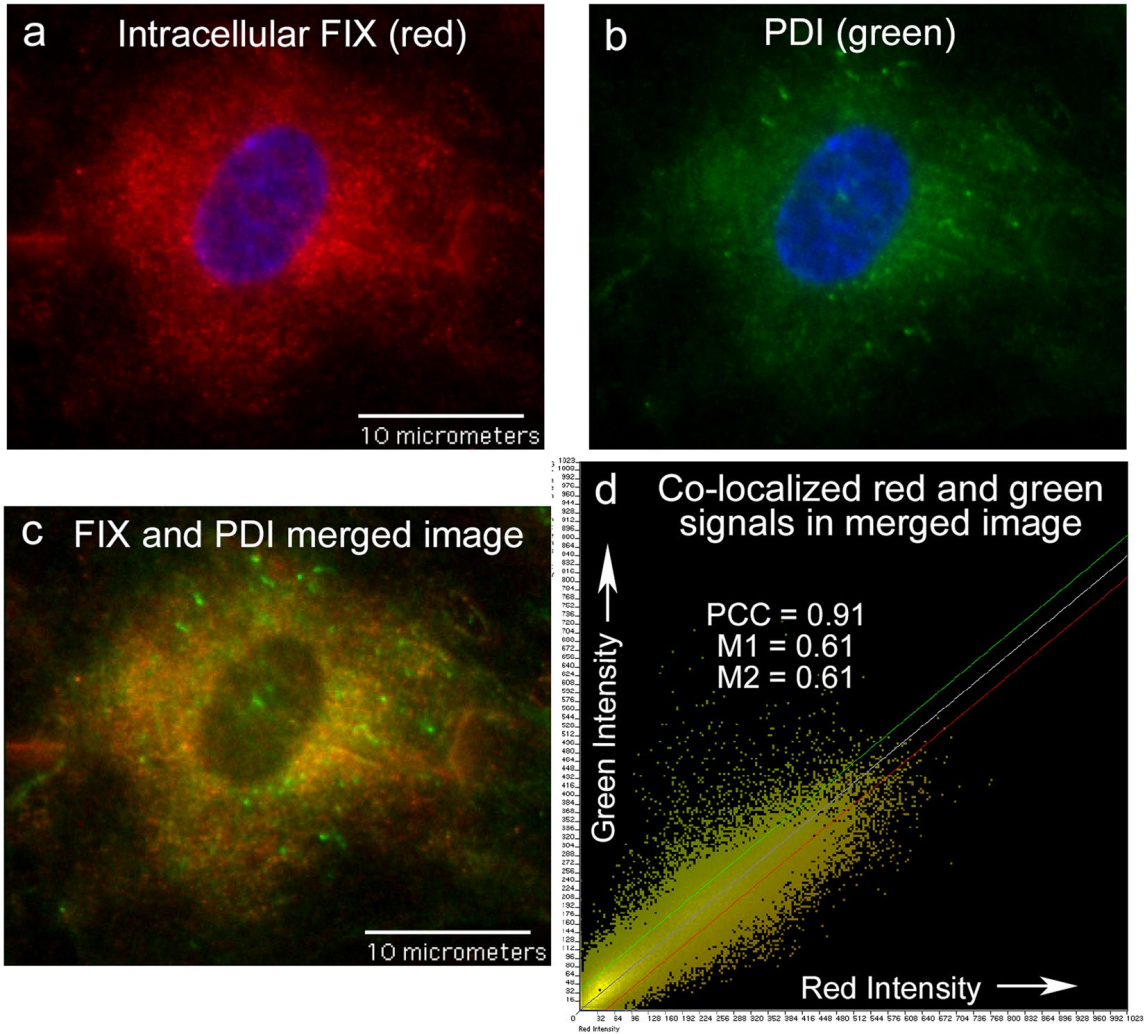
Detection of FIX in HUVEC cytoplasm is associated with the ER. HUVECs were fixed with 1% p-formaldehyde and treated with 0.02% Triton-X to allow intracellular staining, and then stained with sheep anti-human FIX plus secondary donkey anti-sheep IgG-AF-647, followed by mouse anti-protein disulphide-isomerase (PDI) plus goat anti-mouse IgG-AF-488. Cells were imaged at 60X and cell nuclei were detected with DAPI (blue). Panel (**a**): FIX (red); panel (**b**) PDI (ER protein, green); panel (**c**) merged image of FIX (red) and PDI (green); and panel (**d**) the intensity scatter plot of the merged image in (**c**).

**Figure 6:**
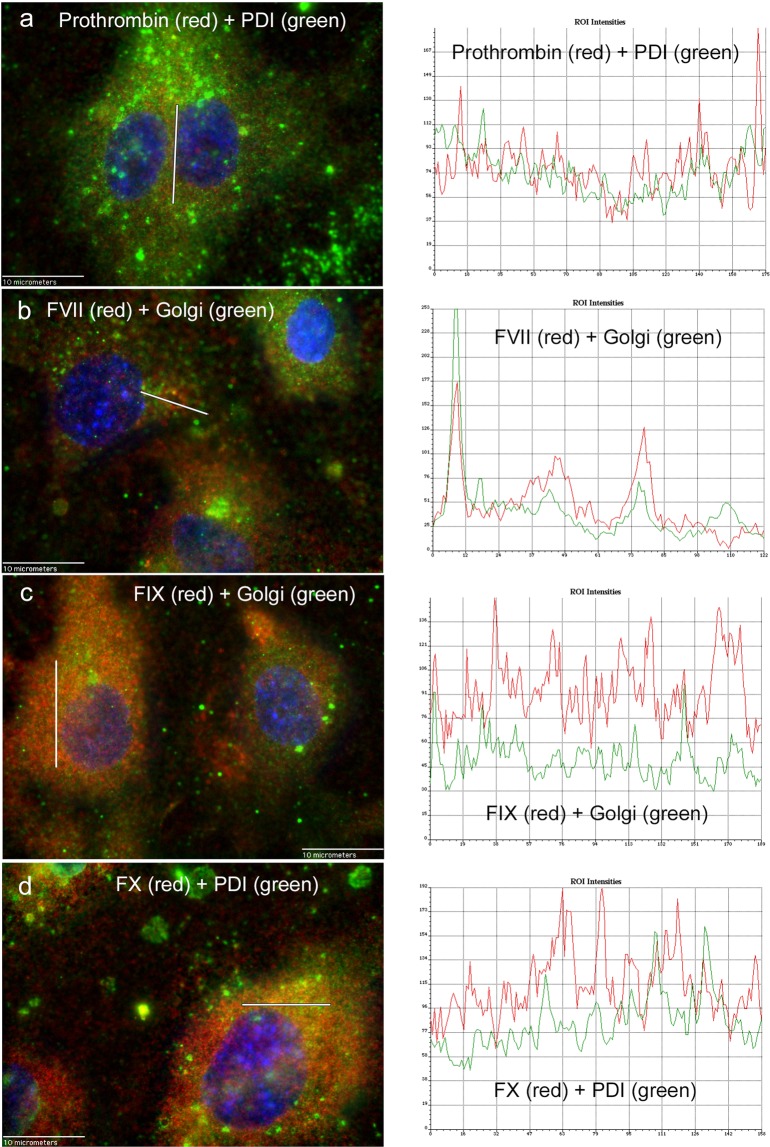
Graphs depicting co-localized detection of intracellular coagulation factors with Golgi and ER proteins. Intensities were measured from red and green channels along a single line in merged images of HUVECs stained simultaneously with antibodies against coagulation proteins and antibodies either to Golgi or PDI. The intensities were measured along the white line shown on the merged images (left panels) and the corresponding graphs (right panels). Fluorescence intensity is on the y-axis and distance (in pixels) is on the x-axis. (**a**) Prothrombin (red) plus PDI (green), line = 10 µm (**b**) FVII (red) plus Golgi (green), line = 7 µm (**c**) FIX (red) plus Golgi (green), line = 11 µm and (**d**) FX (red) plus PDI (green), line = 9 µm. HUVECs were imaged at 100× and nuclei were detected with DAPI. Images were selected from 4–16 similar images for each coagulation protein. The specific detection antibodies are listed in the Fluorescence colocalization measurement section of the Methods. The region of interest (ROI) is the line where the intensities were measured.

An example of FIX detection in HUVECs in which FIX is highly associated with the ER by both location (PCC) and signal overlap (M1 and M2) is shown in Fig. 5. Graphs of red and green fluorescent intensities measured along a single line in merged images of these coagulation factors with either Golgi protein or PDI provides further visual demonstrations of the co-localized proteins in HUVECs (Fig. 6).

In contrast to Golgi and ER proteins, intracellular FIX was not packaged in WPBs with VWF. In merged images of LSECs stained concurrently with FIX and VWF, the average PCC, M1, and M2 values indicate minimal location and signal overlap of VWF in WPBs and cytoplasmic FIX (Table 2). Merged images and dual channel intensity graphs demonstrating the minimal overlapping detection of FIX with VWF in LSECs and HUVECs are shown in Supplementary Fig. S3.

**Table 2:** Negative co-localization of cytoplasmic FIX with VWF.

	FIX and VWF 100×, mean ± SD (range)
PCC	0.29 ± 0.17 (0.01–0.55)
M1	0.35 ± 0.04 (0.26–0.42)
M2	0.40 ± 0.06 (0.27–0.50)
# of cells analyzed	24

Colocalization coefficients, Pearson’s (PCC) and Manders’ (M1 and M2), were measured in LSEC images stained concurrently for FIX and VWF in WPBs using the antibody pairs listed in the Fluorescence colocalization measurements section within the Materials and Methods.

HUVECs were used for co-localization studies with coagulation proteins and ER and Golgi markers because these ECs provide a rapidly growing non-hepatic EC model and have higher FVII, FIX, and FX protein production compared to LSECs and GMVECs. LSECs, our hepatic microvascular EC, were used in the co-localization experiments with FIX and VWF (in addition to HUVECs) because the liver is also a source of coagulation proteins. Fibroblasts were not evaluated in co-localization experiments because they produce lower amounts of FVII, FIX, and FVII than human ECs and have no detectable production of prothrombin.

In summary, the detection data show that EC prothrombin, FIX, FX, and FVII co-localize with ER and Golgi markers, indicating intracellular coagulation factor production. The negative co-localization data are evidence that LSEC-produced FIX is not stored in WPBs with VWF.

In the next experiments we compared the changes in EC and fibroblast coagulation protein production and gene expression in response to inflammatory (tumour necrosis factor [TNF]) and chemical (aurintricarboxylic acid [ATA]) agents.

### Quantification of TF, FVII, FIX, FX, and prothrombin in HUVEC lysates following TNF and ATA exposure

Coagulation protein levels were measured in lysates of untreated HUVECs, and HUVECs exposed to either 10 ng/ml TNF or 30 µg/ml ATA for 24-hours in serum-free media. ATA, a triphenyl-methyl dye, has previously been demonstrated to increase EC expression of *THBD* (gene for TM)^44^. Conversely, the expression levels of *THBD* and *PROCR* (gene for EPCR) are reduced by TNF^45,46^.

**Figure 7:**
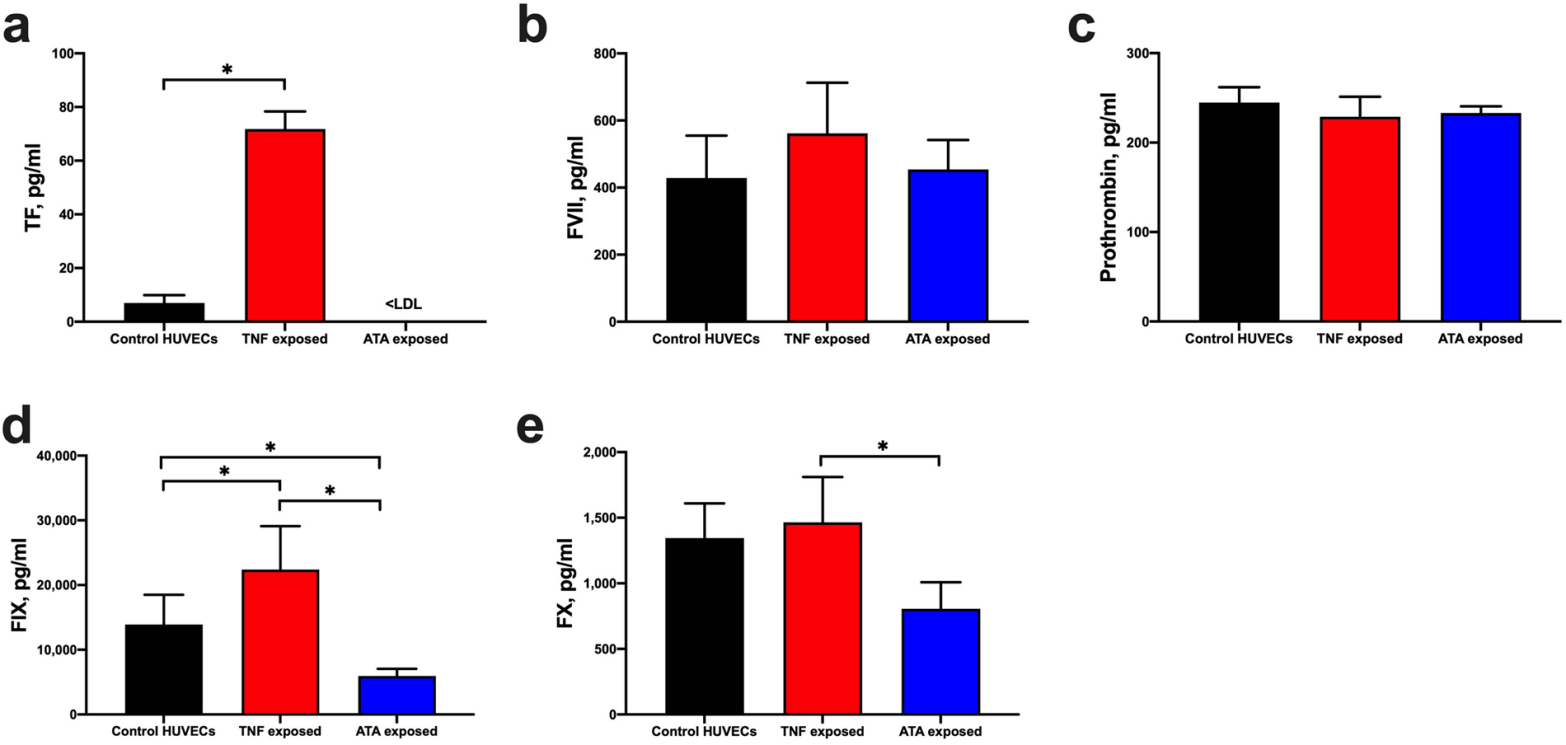
Quantification of TF, FVII, FIX, FX, and prothrombin in HUVEC lysates following TNF and ATA exposure. Cell lysates were prepared from HUVECs after exposure to either 10 ng/ml TNF, 30 µg/ml ATA, or neither for 24-hours in serum-free media for quantification of (**a**) TF, (**b**) FVII, (**c**) prothrombin, (**d**) FIX, and (**e**) FX by ELISA. Concentrations of the proteins (means + SD) for HUVECs (n = 3–8) were normalized to total lysate protein to account for cell number differences. Values below the lowest detectable limit of the assay are noted as <LDL. *p < 0.05.

TNF exposure resulted in a 10-fold increase in HUVEC TF production (p < 0.0001, Fig. 7a) and a 1.6-fold increase in FIX protein concentrations compared to control HUVECs (p = 0.0216, Fig. 7d). Levels of FVII (Fig. 7b), prothrombin (Fig. 7c), and FX (Fig. 7e) were unchanged following TNF or ATA exposure. ATA exposure resulted in a 60% decrease in FIX (p = 0.0455, Fig. 7d), and an undetectable amount of TF. Even after exposure to TNF, HUVECs produced 960-fold less TF than untreated fibroblasts (p < 0.0001, Supplementary Fig. S4).

In summary, HUVECs exposed to TNF had increased TF and FIX protein levels, whereas exposure to ATA reduced FIX and TF protein levels.

### Coagulation protein and regulatory protein gene expression changes in HUVECs and fibroblasts with TNF and ATA exposure

**Figure 8:**
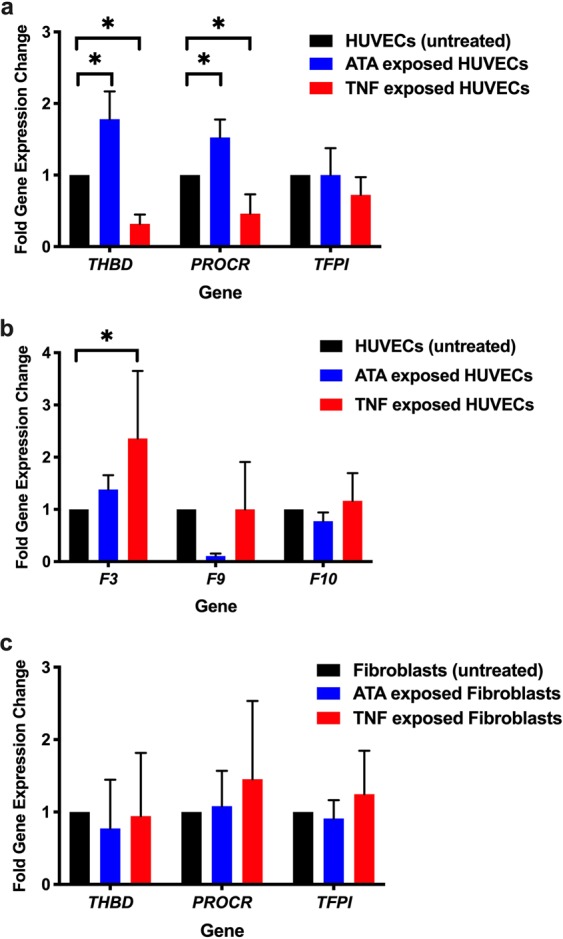
The effect of TNF and ATA exposure on HUVEC and fibroblast expression levels of *THBD, PROCR*, and *TFPI*, and HUVEC expression of *F3, F9*, and *F10*. The changes in gene expression of the surface coagulation regulatory proteins were measured in untreated HUVECs and fibroblasts (black), cells exposed to 10 ng/ml TNF (red), and cells exposed to 30 µg/ml ATA (blue) for 24 hours. Graphs show fold-changes in expression of (**a**) *THBD, PROCR*, and *TFPI* and (**b**) *F3, F9*, and *F10* in HUVECs and (**c**) *THBD, PROCR*, and *TFPI* in fibroblasts (means + SD). Number of gene measurements per experimental condition, HUVECs: *THBD*, n = 6; *PROCR*, n = 4–5; *TFPI*, n = 3; *F3*, n = 3–4; *F9*, n = 4; *F10*, n = 3; for fibroblasts, each gene was measured 4 times under each experimental condition. *p < 0.05.

The changes in *THBD*, *PROCR*, and *TFPI* (genes for TM, EPCR, and TFPI, respectively) expression levels were measured in HUVECs and fibroblasts after exposure to TNF or ATA for 24 hours (Fig. 8a). *THBD* levels were 3-fold lower (p < 0.0001) and *PROCR* levels were 2-fold lower (p = 0.0054) in TNF-exposed HUVECs. In contrast, *THBD* levels increased 1.8-fold (p < 0.0001) and *PROCR* levels increased 1.5-fold (p = 0.0041) in ATA-exposed HUVECs. There was no significant change in *TFPI* expression in HUVECs following 24-hour TNF or ATA exposure.

Expression levels of *F3* (gene for TF), *F9*, and *F10* were also measured in HUVECs following exposure to TNF or ATA for 24 hours. *F3* levels were 2.3-fold higher (p = 0.021) in TNF-exposed HUVECs (Fig. 8b). *F7* expression levels were not consistently detected under any of the experimental conditions. Expression levels of *THBD*, *PROCR*, and *TFPI* were unchanged in fibroblasts after TNF or ATA exposure (Fig. 8c).

In summary, only 3 of the 6 genes studied in HUVECs were affected with exposure to TNF and ATA. HUVECs exposed to TNF had decreased *THBD* and *PROCR* expression, whereas ATA exposure resulted in the opposite (i.e., increased expression of these genes). The only coagulation factor gene affected in HUVECs was *F3* (TF gene), where TNF exposure resulted in an increase in expression. Neither TNF nor ATA changed fibroblast expression levels of these coagulation genes.

In the final experiments, we measured the generation of activated FX on the surfaces of live cultures of ECs and fibroblasts using a chromogenic substrate. The FX activation experiments included measurements after cell exposure to the inflammatory and chemical agents.

### Functional evidence of EC coagulation proteins: FX activation occurs on HUVEC surfaces and is absent on fibroblast surfaces

**Figure 9:**
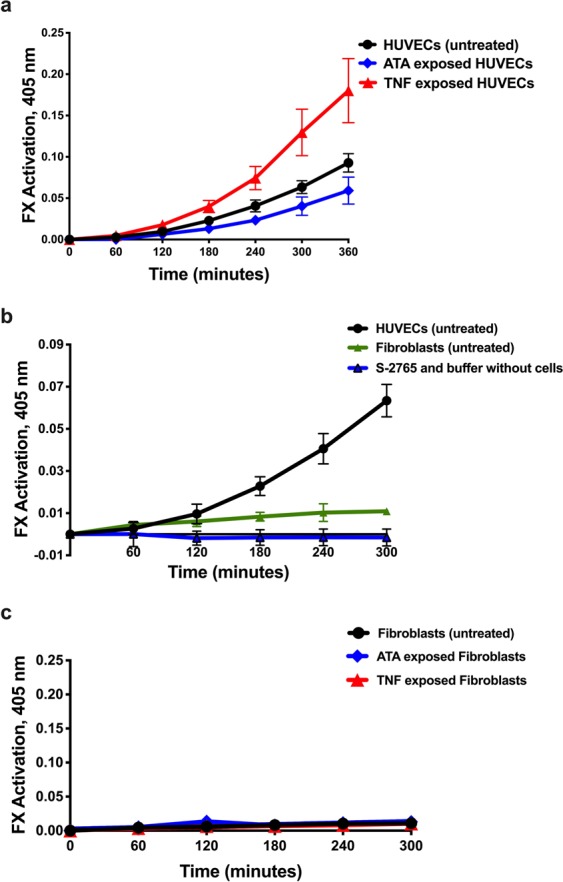
Comparison of FX activation on surfaces of HUVECs and fibroblasts. (**a**) FX activation was measured over time on HUVEC surfaces (in flasks and without addition of exogenous coagulation factors or phospholipids) by monitoring activated FX-mediated cleavage of the chromogenic substrate S-2765. The extent of FX activation was compared in untreated HUVECs (black, n = 11), HUVECs exposed to 10 ng/ml TNF (red, n = 6), and HUVECs exposed to 30 µg/ml ATA (blue, n = 7) for 24 hours. The FX activation in TNF-exposed HUVECs was significantly* higher than untreated HUVECs at time points 180 through 360 min; while ATA-exposed HUVECs generated significantly* less activated FX than untreated HUVECs at time points 240 to 360 min. (**b**) The extent of FX activation generated over time on surfaces of untreated HUVECs (black, n = 11) and fibroblasts (green, n = 4) is shown and compared with the chromogenic substrate S-2765 alone (blue, n = 6), demonstrating the absence of substrate self-hydrolysis. (**c**) FX activation was measured over time on fibroblast surfaces (in flasks and without addition of exogenous coagulation factors or phospholipids). The extent of FX activation was compared in untreated fibroblasts (black, n = 4), fibroblasts exposed to 10 ng/ml TNF (red, n = 4), and fibroblasts exposed to 30 µg/ml ATA (blue, n = 4) in serum-free media for 24 hours. *p < 0.05.

FX was activated on untreated HUVEC surfaces, without the addition of any exogenous coagulation factors or phospholipids. FX activation was measured by the activated FX-mediated cleavage of chromogenic substrate S-2765 (in the presence of a thrombin inhibitor) over a 6-hour period. The levels of FX activation were significantly changed in HUVECs exposed to either TNF (increased FX activation) or ATA (decreased FX activation) (Fig. 9a). Time points of significance between control and TNF-exposed HUVECs were 180 (p = 0.0191), 240, 300, and 360 minutes (p < 0.0001). Time points of significance between control and ATA-exposed HUVECs were 240 (p = 0.0153), 300 (p = 0.0001), and 360 minutes (p < 0.0001). There was no auto-cleavage of the chromogenic substrate under identical experimental conditions measured in parallel using flasks without cells (Fig. 9b). Addition of purified bovine coagulation factors activated FIX and zymogen FX did not result in increased FX activation measured on HUVEC surfaces (Supplementary Fig. S5). Under identical experimental conditions, fibroblasts produced a minimal amount of activated FX. There were significantly higher levels of FX activation generated in HUVECs at 180, 240, and 300 minutes (p < 0.0001) compared to FX activation generated by fibroblasts (Fig. 9b). The extent of FX activation by fibroblasts exposed to either TNF or ATA for 24 hours was unchanged from untreated fibroblasts (Fig. 9c).

In summary, HUVECs activate FX on their surfaces without the addition of external coagulation factors. TNF decreased HUVEC expression of *THBD*, and *PROCR*, and increased *F3* levels while increasing TF and FIX production. The result was an increase of FX activation on HUVEC surfaces. ATA increased *THBD* and *PROCR* expression, and decreased TF and FIX production, resulting in a decrease in HUVEC surface FX activation. Untreated HUVECs activated more FX than fibroblasts. In contrast to HUVECs, TNF and ATA had no effect on FX activation on fibroblast surfaces.

### FX activation on fibroblast surfaces following the addition of FVII and FX proteins

**Figure 10:**
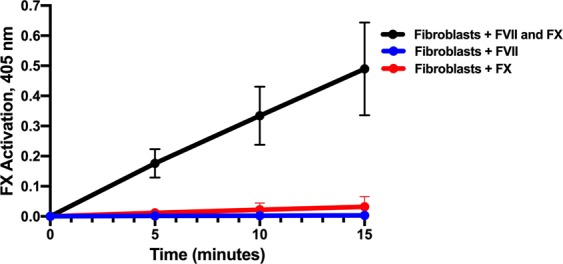
FX activation on fibroblast surfaces following the addition of human FVII and FX proteins. FX activation was measured on fibroblast surfaces by monitoring the hydrolysis of the chromogenic substrate S-2765 over 15 minutes. In a subset of measurements, plasma levels of either FVII (0.7 µg/ml), FX (10 µg/ml), or both were added to the calcium-containing buffer in the fibroblast flasks immediately before the addition of S-2765. There was minimal FX activation following addition of FVII alone (n = 5), increased FX activation following addition of FX alone (n = 5), and a marked increase in FX activation following the addition of both FVII and FX (n = 7) to the fibroblast containing-flasks.

Fibroblasts, despite high levels of TF and evidence of both FVII and FX production, generated relatively low levels of activated FX compared to HUVECs. Because of this result, FX activation was measured on fibroblast surfaces following the addition of plasma levels of either FVII (0.7 µg/ml) or FX (10 µg/ml) alone, or with the addition of both FVII and FX. The FVII and FX proteins, commercially purified from human plasma, were provided as zymogens, not the active forms of the proteins. An immediate and marked increase in FX activation was measured following the addition of both FVII and FX proteins when compared to the addition of either FVII or FX proteins alone. FX activation was significantly higher following FVII and FX addition at 5, 10, and 15 minutes compared to the addition of either FVII or FX alone (p < 0.0001, Fig. 10).

In summary, FX activation was not observed on fibroblast surfaces without the addition of external coagulation proteins. Human fibroblasts differ from HUVECs in that they have sufficiently high TF receptors that can initiate FX activation effectively if FVII and FX are added externally. HUVECs, in contrast to fibroblasts, produce FVIII^18^ and initiate FX activation using only EC-produced coagulation proteins.

### Estimated levels of activated FX on HUVEC and fibroblast surfaces

The levels of activated FX generated on HUVEC and fibroblast surfaces were estimated by measuring the cleavage rate of S-2765 using known concentrations (U/ml) of purified, human activated FX. The activated FX level for each experimental condition was determined by interpolation of measured experimental rates from the curve produced by plotting the known activated FX concentrations against the corresponding cleavage rates (Table 3).

**Table 3:** Estimated levels of activated FX on HUVEC and fibroblast surfaces.

	Rate*	Activated FX, U/ml
HUVECs, untreated	4.348 × 10^–4^	1.245 × 10^−3^
HUVECs, TNF	8.806 × 10^−4^	2.404 × 10^−3^
HUVECs, ATA	2.992 × 10^−4^	8.277 × 10^−4^
Fibroblasts, untreated	3.545 × 10^−5^	<LDL
Fibroblasts, TNF	3.146 × 10^−5^	<LDL
Fibroblasts, ATA	3.701 × 10^−5^	<LDL
Fibroblasts + FVII	2.184 × 10^−4^	5.384 × 10^−4^
Fibroblasts + FX	2.138 × 10^−3^	5.359 × 10^−3^
Fibroblasts + FX + FVII	3.254 × 10^−1^	1.648 × 10^−1^

Using a curve generated by measuring the rates of known concentrations (U/ml) of activated FX, the experimentally measured FX cleavage rates were used to estimate the levels of activated FX generated on cell surfaces.

*Measured cleavage rate of chromogenic substrate S-2765; the increase in absorbance per minute at 405 nm.

LDL: values were below lowest detectable limit of the assay.

## Discussion

**Figure 11:**
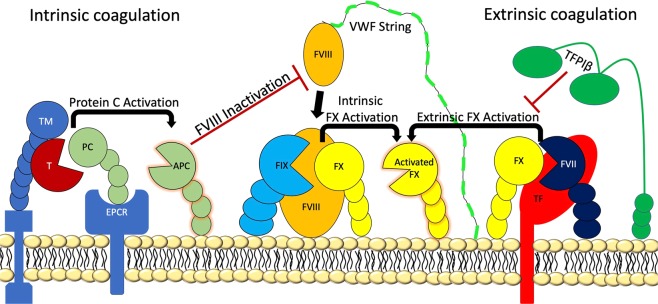
Visual representation of intrinsic and extrinsic coagulation on EC surfaces. VWF strings are secreted from EC WPBs with FVIII attached. FVIII detaches from the VWF strings to form intrinsic tenase (FVIII-FIX) complexes with membrane bound FIX and subsequently activating FX. APC, activated by TM-bound thrombin (T), suppresses EC surface intrinsic FX activation by inhibiting FVIII (and FV). The amount of APC available is dependent on the presence and activity of the surface regulatory proteins, TM (with bound T) and EPCR. TF-bound FVII activates FX, and this reaction is limited by TFPIβ (the predominant isoform of TFPI on the endothelium^68^).

We found that human ECs (HUVECs, GMVECs, and LSECs) produced FX and the coagulation proteins that form the intrinsic (FVIII^12,14–16,18^-FIX) and extrinsic tenase (TF-FVII) complexes in addition to prothrombin. These coagulation factors were produced in forms capable of participating in FX activation on HUVECs without the addition of external coagulation proteins, proteolytic enzymes, or phospholipids. In addition to EC lysates, FVII, FIX, and FX proteins were also detected released into EC supernatants. These results may demonstrate a possible extra-hepatic source of plasma coagulation proteins. A visualization of this EC-based model of coagulation is shown in Fig. 11. Our findings suggest that both intrinsic and extrinsic coagulation reactions can occur on EC surfaces using EC-produced coagulation proteins.

In contrast, FX activation on fibroblast surfaces was considerably lower without the external addition of coagulation protein FX. This occurred although fibroblast and HUVECs lysates have similar concentrations of FX. Fibroblast supernatants, however, have ~6-fold higher FX concentrations than HUVEC supernatants. It is possible that fibroblasts bind FX inefficiently or have relatively fewer available binding sites for FX. This would explain why fibroblasts required external FX supplementation for extrinsic FX activation to occur on the high TF-bearing fibroblast surfaces. It may also explain why fibroblast supernatants have higher concentrations of FX than is present in cell lysates. Extrinsic activation of FX on fibroblasts may occur *in vivo* following vascular injury and fibroblast exposure to flowing blood (containing FVII and FX).

The liver is the largest internal organ containing ~60% hepatocytes and ~35% LSECs + stellate cells. Hepatocytes are estimated to be only 0.8% of the total cells in humans^47,48^, while vascular EC numbers are calculated to be from 6 × 10^11^ to 2.5 × 10^12^ and ~2.1% of all human cells^47,49^. Although the liver has long been considered to be the site of most (or all) coagulation factor production, ECs are now known to be the primary cellular source of FVIII in mice and humans^10–18^. Our results show that coagulation factors in addition to FVIII are produced in several types of ECs, and detectable in EC supernatant (FVII, FIX, and FX) implying that the role of EC-produced coagulation factors in hemostasis may have been underestimated.

In addition to FX, ECs have previously been shown to synthesize and bind FV on their surfaces^50,51^. This suggests that ECs may also be capable of assembling active prothrombinase (FV-FX) complexes for the activation of prothrombin to thrombin. In our experiments, prothrombin was detected in the lysates of HUVECs, GMVECs, and LSECs, and intracellular prothrombin was found to be associated with Golgi and ER proteins in ECs (Table 1). Previously, HUVEC generated prothrombin activation products, meizothrombin and thrombin, have been demonstrated to activate PC^52^. The chromogenic substrate S-2765 used to measure FX activation on EC surfaces contained a thrombin inhibitor (to prevent any unwanted thrombin cleavage of S-2765). However, the generation of thrombin; thrombin-TM binding; thrombin-dependent activation of PC to APC; and inhibition of FVIII (and FV) could occur during the 24-hours of experimental incubation (in serum-free media) prior to the addition of the thrombin inhibitor-containing FX S-2765 substrate.

FX activation on HUVEC surfaces after exposure to TNF was >90% higher than in untreated HUVECs (Fig. 9a). The increase in activated FX was not the result of increased FX protein production following TNF exposure, because FX protein levels measured in lysates of untreated HUVECs were not significantly different than FX levels in TNF-exposed HUVECs (Fig. 7e). In contrast, the higher level of activated FX is likely to be secondary to the increased activity of both intrinsic and extrinsic tenase complexes on HUVEC surfaces in the presence of TNF. Our putative explanation of these concurrent processes is summarized in the following paragraphs.

TNF exposure increases HUVEC expression of *F3* (Fig. 8b) and TF production (Fig. 7a), compatible with increased extrinsic tenase complex formation and activity. In parallel, TNF decreases *THBD* and *PROCR* expression (Fig. 8a), resulting in reduced numbers of surface TM and EPCR receptors, decreased PC activation^46^, and diminished FVIII inactivation^53^. We have previously demonstrated that FVIII is synthesized in human ECs (including HUVECs), stored in EC WPBs, and secreted from ECs bound to ULVWF strings^18^. These processes provide the FVIII necessary for intrinsic tenase complex formation. The increased FVIII availability associated with TNF-suppression of PC activation combined with the higher levels of FIX protein induced by TNF (Fig. 7d) enables more intrinsic tenase complex formation to generate higher levels of activated FX.

In contrast to TNF, HUVECs exposed to ATA generated ~35% less activated FX compared to untreated HUVECs. These lower levels of activated FX may result from decreased intrinsic tenase complex formation due to lower FIX levels (Fig. 7d), and lower amounts of active FVIII. The increase in *THBD* and *PROCR* expression with ATA exposure (Fig. 8a) results in increased TM activity^54^, more APC and quicker inactivation of free FVIII^55^. FIX-bound FVIII, however, is protected from inactivation by APC^56,57^. ATA exposure did not change the gene expression levels of either *F3* or *TFPI* (Fig. 8). However, ATA exposure did decrease protein production of TF, and this could contribute to a reduction in extrinsic-mediated FX activation (Fig. 7a).

Prothrombin, FVII, FIX, FX, and PC (in addition to PS, a cofactor for PC) are vitamin K-dependent proteins and function effectively when attached to cell membranes through calcium binding to their gamma-carboxyglutamic acid residues. Gamma-glutamyl carboxylation of these proteins is likely to occur prior to their release from ECs because prothrombin, FVII, FIX, FX, and PC were able to function in the presence of calcium on the cell surfaces. HUVECs contain endogenous PC, and PC activation on HUVEC surfaces without the addition of exogenous PS has previously been demonstrated^19,46^. We have also provided evidence that HUVECs produce prothrombin (in untreated and TNF/ATA treated cells) to allow for the thrombin-mediated activation of PC. All proteins and cofactors necessary for coagulation factor activation and activity were present within ECs, as there was no addition of external proteins.

Previous work has shown that the inflammatory cytokine TNF induces procoagulant activity in human ECs^58,59^, and that FIX plays a critical role in FX activation and thrombin formation in TNF-associated thrombosis^60^. Our experiments demonstrating increased FX activation on EC membranes following exposure to TNF provide additional insight into basic mechanisms of disseminated intravascular coagulation during infection. Specifically, increased TNF in sepsis may be associated with increased EC *F3* expression and TF production, as well as down-regulation of *THBD* and *PROCR*, resulting in decreased APC generation for suppression of FVIII and FV inactivation. These TNF-mediated changes may result in increased intrinsic and extrinsic coagulation activity on EC surfaces.

In patients with hemophilia A and hemophilia B, the EC extrinsic (TF-FVII) tenase complex must be quantitatively inadequate to compensate for the low level of either FVIII or FIX. This inadequacy can be overcome in some hemophilia A patients (e.g., those with antibodies to FVIII) using therapeutic augmentation of the extrinsic (TF-FVII) tenase pathway by intravenous FVII or subcutaneous concizumab, a monoclonal antibody against TFPI^61^. These therapies may function, at least in part, on the surfaces of TF-containing ECs or fibroblasts.

In conclusion, our findings demonstrate that multiple types of human ECs produce the proteins required for FX activation by the intrinsic and extrinsic coagulation pathways. The model HUVEC system is capable of propagating coagulation reactions on their cell surfaces in a tightly regulated manner without the addition of external coagulation proteins, proteolytic enzymes, or phospholipids. Our study provides new information on possible EC (and fibroblast) involvement in haemostasis and thrombosis.

## Materials and Methods

### Human endothelial cells

Pooled primary human HUVECs (200p-05n) were purchased from Cell Applications Inc. GMVECs (ACBRI-128 V) and LSECs (ACBRI-566 V) were isolated from single donors and purchased from Cell Systems. Basic EC growth media MCDB-131 (Sigma-Aldrich) plus penicillin/streptomycin/L-glutamine/amphotericin (PSQA, Life Technologies) was augmented with low serum growth supplement (S003K, fetal bovine serum [FBS] concentration 2% v/v) for HUVECs and microvascular growth supplement (S00525, FBS concentration 5% v/v) for LSECs and GMVECs (Life Technologies). ECs were non-enzymatically removed from tissue culture flasks using 5 mM EDTA in Ca^+2^, Mg^+2^-free PBS and cell scraping. Each EC type was used in experiments at passages 2–8 derived from a single lot.

### Human dermal fibroblasts

Single donor human dermal fibroblasts were purchased from Zenbio (DF-F) at passage 2. Fibroblasts were grown in DMEM (Corning 10–013-CM) with 10% FBS (Biowest S1620) plus PSQA and used in experiments at passages 3–8.

### EC and fibroblast exposure to TNF or ATA

To determine the effect of cytokine and chemical exposure on gene expression, coagulation factor production, and surface FX activation, cells were washed with PBS before 24-hr exposure to either serum-free media alone (MCDB-131 + insulin-transferrin-selenium, Life Technologies), 10 ng/ml TNF, or 30 µg/ml ATA in serum-free media.

### Reverse transcription real-time (RT) quantitative polymerase chain reaction (qPCR)

RNA was isolated using TRIzol, chloroform extraction, and isopropanol precipitation. RNA integrity was verified by 1%-agarose-formaldehyde electrophoresis and 260/280 optical ratios. SuperScript VILO MasterMix (Invitrogen) was used to reverse-transcribe the RNA. The resulting cDNA samples (200 ng) were amplified in triplicate by RT-qPCR under the conditions: 95 °C for 3 min, 50 cycles of (10 sec at 95 °C, 10 sec at 55 °C, 30 sec at 72 °C), and 95° for 10 sec (CFX96, BioRad). The amplified DNA products were analyzed using TaqMan Gene Expression Assays (with 6-carboxy-fluorescein-labeled probes that span target exon junctions, Supplementary Table S1) with PerfeCT FastMix II (Quanta).

### Protein measurements

#### Endothelial cell and fibroblast lysates

Cell lysates were prepared from HUVECs, GMVECs, LSECs, and fibroblasts grown in T-75 flasks until confluence in appropriate complete media. To ensure that all traces of exogenous proteins were eliminated, after removing the media, the cells were washed 3X with 10 ml of cold, sterile Tris buffer (50 mM Tris, pH 7.3, 1% BSA, and 5 mM CaCl_2_). Cells were then lysed with 500 µl of CelLytic M (ice cold, Sigma-Aldrich C-2978) + 10 µl of Halt protease/phosphatase inhibitor cocktail (Thermo Scientific, 78430) for 15 min with rocking. Lysed cells were collected with a cell scraper, placed into a chilled tube and centrifuged at 12,000 g for 15 min at 4 °C. The supernatants were collected and stored at -80 °C until assays for prothrombin, TF, FVII, FIX, FX, and total protein.

In a subset of confluent HUVEC-containing flasks, 24-hours prior to lysate preparation, the cells were washed 3X with Ca^+2^, Mg^+2^-containing PBS and the media was replaced with serum-free medium. HUVECs were maintained for 24 hours in serum-free medium alone, or with either 10 ng/ml TNF or 30 µg/ml ATA in serum-free media prior to lysate collection for quantification of prothrombin, TF, FVII, FIX, and FX.

### Quantification of prothrombin, TF, FVII, FIX, and FX in EC and fibroblast lysates

To determine if ECs and fibroblasts produce coagulation proteins, the concentrations of prothrombin, TF, FVII, FIX, and FX protein were quantified in GMVEC, LSEC, HUVEC, and fibroblast lysates. Proteins were quantified in undiluted lysates (except for TF in fibroblast lysate which was diluted 1:200) using Abcam assays for TF (ab220653), FVII (ab168545), FIX (ab108831), and FX (ab108832), and LifeSpan BioSciences assay for human prothrombin/thrombin (LS-F36128). The antibodies in these kits only detect human TF, FVII, FIX, FX, and thrombin/prothrombin proteins, respectively. There is no cross-reactivity with bovine proteins and the presence of 10% FBS in culture media will not affect the assays. The lower sensitivity limits of the ELISA systems used were: TF: 3.6 pg/ml, FVII: 0.15 ng/ml, FIX: 1.563 ng/ml, FX: 0.781 ng/ml, and prothrombin: 0.188 ng/ml. The FVII assay includes a positive plasma control and a low plasma control, in addition to human FVII calibration standards. The low control concentration was 0.5 ng/ml, and each cell lysate sample with detectable FVII levels had FVII concentrations above the low plasma control. The FIX assay was calibrated against the NIBSC/WHO purified Human FIX preparation 07/182. The TF assay is specific to the full-length, extracellular portion of human TF. Values obtained for TF, FVII, FIX, FX, and prothrombin were normalized to total lysate protein (to account for cell number differences) determined by the Bradford method. Total average protein (mg/ml ± standard deviation [SD]) in each EC type was: GMVECs 5.8 ± 0.2; LSECs 6.2 ± 0.4; HUVECs 5.9 ± 1.0; and 5.3 ± 0.3 in fibroblasts.

### Quantification of FVII, FIX, FX, and prothrombin released into EC and fibroblast supernatant

To determine if ECs and fibroblasts release coagulation proteins into the media, GMVECs in T-25 flasks and LSECs, HUVECs, and fibroblasts in T-75 flasks, were grown to 100% confluence. Cell were then washed 3X with Ca^+2^, Mg^+2^-containing PBS and the media was replaced with serum-free medium for 24 hours (350 µl per T-25 flask and 1.5 ml per T-75 flask). Following 24 hours, the serum-free media was collected into tubes containing 20% BSA to produce a final concentration of 1% BSA (17.5 µl per T-25 flask and 75 µl per T-75 flask). Levels of FVII, FIX, FX, and prothrombin were quantified in cell supernatant using the same commercial assays that were used to quantify levels in cell lysates as described above: FVII (ab168545), FX (ab108832), and prothrombin (LS-F36128). The concentration of FIX in cell supernatant was quantified using the Abcam kit ab188393, which has a minimal detectable FIX level of 0.230 ng/ml and the detection antibodies do not cross-react with bovine FIX.

### Fluorescent microscopy studies

#### Microscope image acquisition

The microscope system consists of a Nikon Diaphot TE300 microscope equipped with CFI Plan Fluor 60× oil, numerical aperture (NA) 1.4 and CFI Plan Apo Lambda 100× oil, NA 1.45 objectives, a 10× projection lens (Nikon, Garden City, NY) and a Prior motorized stage. Fluorescent images were obtained with a SensiCamQE CCD camera (Cooke Corp., Romulus, MI) using dual filter wheels (Prior) with single band excitation and emission filters for FITC/TRITC/CY5/DAPI (Chroma, Rockingham, VT). Cell images were processed and analyzed using IP Lab software version 3.9.4r4 with a fluorescence colocalization module (Scanalytics, Inc., Fairfax, VA). Images acquired using the 60× objective have dimensions of 78 µm × 58 µm and images acquired using the 100× objective are 41 µm × 30 µm. Calibration bars on images are 10 µm.

### Cell intracellular fluorescent staining

To determine if coagulation proteins are present in the cytoplasm of LSECs, GMVECs, and HUVECs, cells were grown on glass coverslips (1.5-mm thick) pre-coated with gelatin and were washed with PBS 3X at each step. Washed cells were fixed with 1% p-formaldehyde in PBS and followed by 0.02% Triton-X to enable intracellular staining. The cells were stained for 15 min with each primary (diluted 1:100 in PBS containing 1% BSA) plus fluorescent AF-labeled secondary antibody at 20 µg/ml (Life Technologies). Primary antibodies purchased through Haematologic Technologies include: sheep anti-human FIX (PAHFIX-SAP), sheep anti-human FX (PAHFX-S), sheep anti-human FVII (PFVII-S), and sheep anti-human prothrombin (PAHFII-SAP) plus secondary donkey anti-sheep IgG-AF-647. Additional primary antibodies that were used are: rabbit anti-human VWF (Ramco Laboratories) plus chicken anti-rabbit IgG-AF-488; and mouse anti-human FVIII (ThermoFisher, F8-5.5.72) plus goat anti-mouse IgG-AF-488. Cell nuclei were detected with DAPI (4′,6-diamidino-2-phenylindole, 1.5 µg/ml) that was included in the mounting medium (Fluoro-Gel II, Electron Microscopy Sciences).

### Fluorescence colocalization measurements

To provide additional evidence of coagulation protein synthesis in ECs, intracellular coagulation proteins prothrombin, FVII, FIX, and FX were detected in ECs simultaneously with proteins specific for the endoplasmic reticulum (ER) and Golgi using primary antibodies combined with fluorescently labeled secondary antibodies. The entire merged image was analyzed for red and green signal similarities in shape, location, and intensity. Each coagulation protein was detected using polyclonal sheep anti-human antibodies plus donkey anti-sheep IgG-AF-647 (designated as the red channel). Detection of each coagulation proteins was compared for degree of colocalization with the ER structural marker, protein disulphide-isomerase (PDI), (mouse anti-PDI, clone RL90, abcam2792-100, plus goat anti-mouse IgG-AF-488, green channel) and Golgi marker (rabbit anti-human 58kD Golgi protein, abcam5820, plus chicken anti-rabbit IgG-AF-488 or with donkey anti-rabbit-594), both designated as the green channel.

In some experiments ECs were stained to analyze VWF co-localization with FIX. The purpose of co-localization experiments with FIX and VWF was to determine if FIX is stored in the same intracellular location as FVIII, which is stored in association with VWF in EC WPBs^18^. VWF was detected using rabbit anti-human VWF + secondary chicken anti-donkey IgG-AF-488 (green channel designation). Preceding colocalization analysis, the cell images were processed for empirical background subtraction. Fluorescence cross-talk and bleed-through were minimized by selecting fluorophores on secondary detection antibodies with non-overlapping spectra paired with narrow bandwidth filters, as previously described^18^.

The degree of similarity between detection in the red and green channels in merged images was determined using the fluorescence colocalization module within IP Lab software (Scanalytics, Inc.). The colocalization module calculates values for Pearson’s correlation coefficient (PCC), that contributes shape information based on intensity distribution, and Manders’ coefficients (M1 and M2), that describes the frequency that the red and green signals are detected in the same location throughout the merged image^62–65^. A thorough discussion of the mathematical theory has been reported by Comeau, *et al*.^63^. Because PCC and M1 and M2 are determined in different mathematically ways, a positive calculated value (0.5 or higher) for either PCC or M1 and M2 indicates colocalization^64,65^. Intensity scatter plots can also visualize the extent of signal co-localization. A merged image with equal red and green intensities and perfect distribution will result in a scatter plot with a centered line with a PCC = 1^64,65^.

In select merged images detecting coagulation factors prothrombin, FVII, FIX, and FX with either PDI or Golgi proteins, dual channel intensities were measured along a 0.058 µm width line (1 pixel) with lengths ranging from 7–11 µm in images obtained at 100×. Plots of the green intensity values (PDI or Golgi) and the red intensity values (coagulation proteins) at the same locations allowed a visual demonstration of signal colocalization. Similar methodology has been previously demonstrated as a measure of intracellular protein co-localization^66^. This same analysis was used to show negative co-localized detection of intracellular FIX with VWF in both LSECs and HUVECs. HUVECs were used as an EC model for co-localization experiments as they produce the largest quantities of FVII, FIX, and FX proteins compared to LSECs and GMVECs. Co-localization was not done in fibroblasts because of the relatively low amounts of FVII and FIX produced compared to ECs (and undetectable prothrombin production). Co-localization experiments were performed in LSECs because the liver is a major site of coagulation protein production.

### Quantitative gene expression measurements

Expression level changes of coagulation proteins and surface protein genes were quantified to determine the effect of inflammatory cytokine and chemical exposure on HUVECs and fibroblasts. Gene expression changes in *THBD, PROCR*, and *TFPI*, with/without exposure to TNF or ATA, were measured in both HUVECs and fibroblasts, and level changes of *F7, F9, F10*, and *F3* were measured in only HUVECs. HUVECs (7–10 days post-seeding) and fibroblasts (5–8 days post-seeding) were incubated in serum-free media alone, 10 ng/ml TNF or 30 µg/ml ATA in serum-free media for 24 hours prior to RNA extraction. *GAPDH* was used as the reference gene.

Quantitative changes in the gene expression of *THBD, PROCR*, and *TFPI* in HUVECs (as well as *F9, F10*, and *F3*) and fibroblasts exposed to TNF or ATA were calculated using the process developed by Pfaffl, *et al*.^67^. In this procedure, specific primer efficiencies (E) are used to evaluate the amount of cDNA amplification per each PCR cycle^67^. Gene efficiencies were determined by amplification of 100 ng-0.01 ng of cDNA and calculating the slope of the line after plotting the quantification cycle (C_q_) versus ng of cDNA (Supplementary Table S1). Due to the low expression levels of *F9*, amplification products of diluted cDNA with <100 ng were undetectable, the efficiency could not be calculated, and the estimated value of 2 was used. Detection of *F7* expression levels was below the sensitivity limits of the system used.

### Measurement of FX activation on HUVEC and fibroblast surfaces

Experiments measuring FX activation on HUVEC and fibroblast surfaces without the addition of external coagulation proteins were performed to determine if EC and fibroblast-produced coagulation proteins are active on local cell surfaces. HUVECs were used as a model EC subtype for FX activation experiments because of their high level of coagulation protein production compared to the other ECs studied. HUVECs (passages 2–4) and fibroblasts (passages 3–8) were grown in T-75 flasks in serum-containing media until reaching confluence (HUVECs 7–10 days and fibroblasts 5–8 days). 24-hours prior to experimentation, the cells were washed 3X with Ca^+2^, Mg^+2^-containing PBS and the media was replaced with serum-free medium alone, or with either 10 ng/ml TNF or 30 µg/ml ATA in serum-free media.

For FX activation measurements, cells in T-75 flasks were washed 3X with PBS and then either 1 ml of PBS, or 50 mM Tris, pH 7.3 buffer, both containing 1% BSA and 5 mM Ca^+2^ was added, followed by 500 µl of the activated FX chromogenic substrate, S-2765 plus thrombin inhibitor, I-2581 (Diapharma). Two 100 µl samples were removed directly after substrate addition for analysis. Cells were maintained at 37 °C during the experiment, with duplicate samples analyzed every 60 minutes over a 300- or 360-minute time period. Absorbance was measured at 405 nm and 490 nm in a Tecan Infinite M200 Pro plate reader at each time point and values of duplicate samples from the same time point were averaged. The A_490_ was subtracted from the A_405_ to correct for differences in the microplate wells. The activated FX chromogenic substrate composed of S-2765 (15.4 mg) and synthetic thrombin inhibitor, I-2581 (0.4 mg) was reconstituted prior to experiments with 12 ml of sterile water.

### Measurement of FX activation on fibroblast surfaces following the addition of external FVII and/or FX

Human FVII and FX, the proteins required for the extrinsic activation of FX, were added to fibroblasts (which contain high levels of TF) to determine if TF-initiated coagulation can occur on fibroblasts (which, unlike HUVECs, do not initiate coagulation using only fibroblast-produced proteins). In some fibroblast experiments, human coagulation proteins FVII (0.7 µg/ml, HCVII-0030) and FX (10 µg/ml, HCX-0050), both purchased through Haematologic Technologies, were added either alone or together to the calcium-containing buffer immediately before the addition of S-2765. These FVII and FX proteins are the inactive precursors to activated FVII and FX, respectively. Following FVII and/or FX protein and S-2765 addition, duplicate samples were collected every five minutes for analysis as described above.

### Estimation of activated FX on HUVEC and fibroblast surfaces

Activated FX (HCXA-0060, Haematologic Technologies) was initially diluted to 1 U/ml in 50 mM Tris, pH 7.3, buffer containing 1% BSA and 5 mM Ca^+2^. One U was defined as the FX activity contained in 1 ml of normal human plasma. In kinetic assays, rates were measured from 20 concentrations of known activated FX values ranging from 1 U/ml to 9.8 (10^−5^) U/ml. The volume ratio of activated FX (in the Tris buffer) to the chromogenic substrate S-2765 was the same as in the experiments measuring FX activity on cell surfaces. The cleavage rates were determined by mixing 70 µl of each activated FX concentration (U/ml) with 30 µl of S-2765 and then measuring the absorbance at 405 nm at 10 min intervals for 2 hours. The calculated slopes (cleavage rates) were plotted against the FX activities (U/ml). The slopes calculated from data measured in HUVEC and fibroblast experiments were used to calculate the levels of activated FX from the generated curve. The cleavage rate of each known activated FX dilution was measured 4 times. In activated FX samples with activities >0.125 U/ml, cleavage rates were measured over 16 min at 2 min intervals.

### Statistical analysis

GraphPad Prism v 8 software (GraphPad Software Inc., San Diego, CA) was used to calculate standard deviations (SD) and significance of differences between quantitative gene expression changes, protein measurements, ELISA analysis, and in FX activation measurements using one- and two-way ANOVAs and Tukey’s and Dunnett’s multiple comparison tests with an alpha value of 0.05. The SD values in the fluorescent colocalization measurements were calculated using Microsoft Excel.

## Supplementary information

Supplementary Information.

## Data Availability

The datasets generated during and/or analyzed during the current study are available from the corresponding author on reasonable request.
