# Supplementary material for: Production and control of coagulation proteins for factor X activation in human endothelial cells and fibroblasts
Source: Sci Rep. 2020 Feb 6;10:2005. doi: 10.1038/s41598-020-59058-4 (PMC7005260; doi:10.1038/s41598-020-59058-4)
Supplement: Supplementary file 1 — Supplementary Information. [file 41598_2020_59058_MOESM1_ESM.pdf]

Production and control of coagulation proteins for factor X activation in human endothelial cells and fibroblasts

Clay T. Cohen, Nancy A. Turner, Joel L. Moake

Supplementary Information

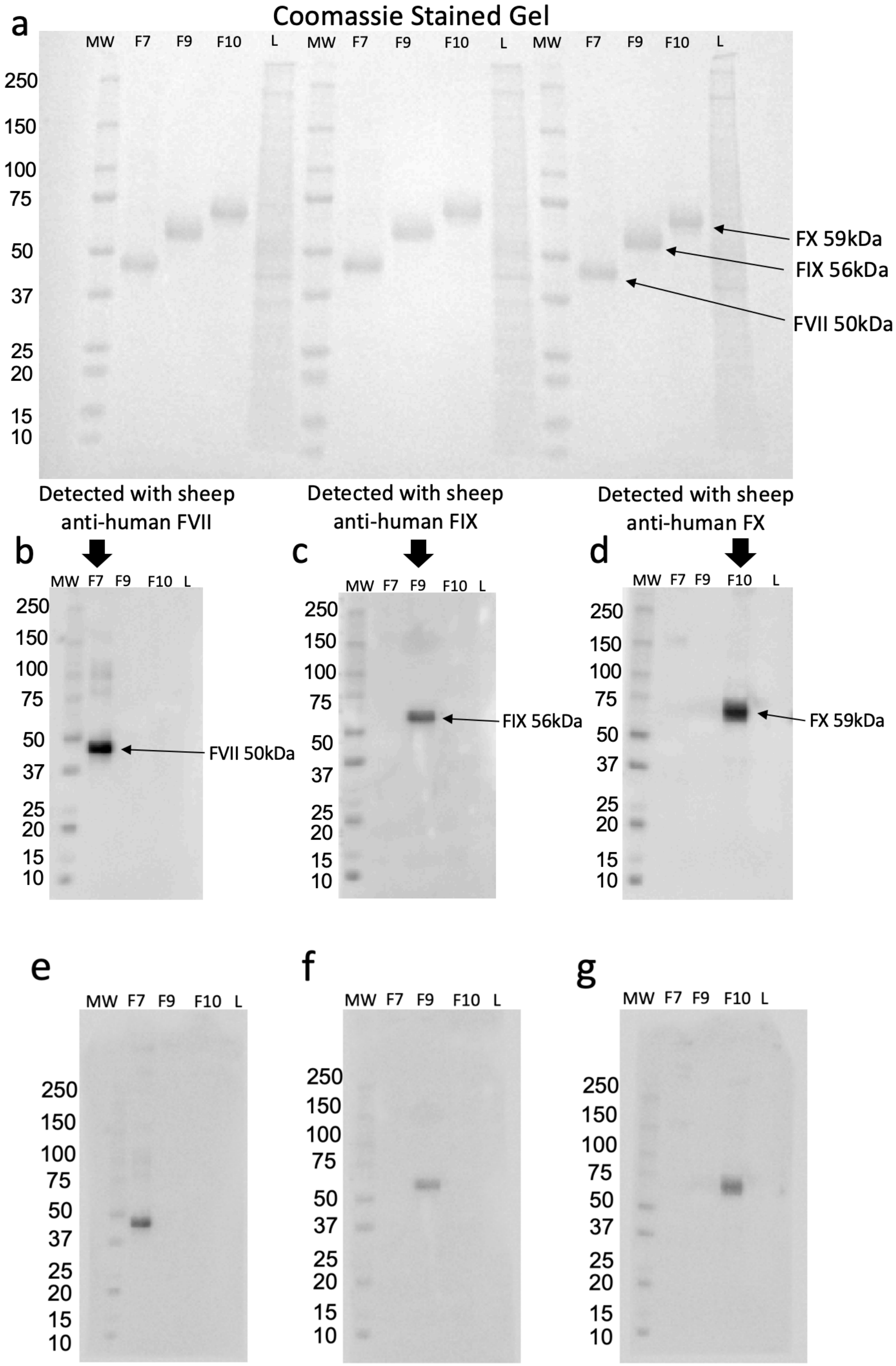
a
Coomassie Stained Gel
MW F7 F9 F10 L MW F7 F9 F10 L MW F7 F9 F10 L
250
150
100
75
50
37
25
20
15
10
FX 59kDa
FIX 56kDa
FVII 50kDa
Detected with sheep anti-human FVII
Detected with sheep anti-human FIX
Detected with sheep anti-human FX
b
MW F7 F9 F10 L
FVII 50kDa
c
MW F7 F9 F10 L
FIX 56kDa
d
MW F7 F9 F10 L
FX 59kDa
e
MW F7 F9 F10 L
f
MW F7 F9 F10 L
g
MW F7 F9 F10 L

Fig. S1 Specificity of antibodies used to detect FVII, FIX, and FX. Denatured, non-reduced samples (1.5μg per lane) of plasma-purified human FVII (F7), FIX (F9), and FX (F10), and GMVEC lysates (L, 2μg per lane) were separated by 4-15% sodium dodecyl sulfate (SDS)-PAGE. MW indicates molecular weight markers in kDa. (a) Full Coomassie stained gel with arrows to indicate the 50-kDa band for FVII, the 56-kDa band for FIX, and the 59-kDa band for FX. (b-d) After the gel was transferred to the PVDF membrane, the membrane was cut into 3 sections. The blot sections, each with MW standards, the 3 coagulation proteins and GMVEC lysates, were incubated with one of the three primary sheep antibodies to human FVII, FIX, or FX, followed by donkey anti-sheep-HRP and chemiluminescent detection. Blot sections were detected in (b,e) with sheep anti-human FVII, in (c,f) with sheep anti-human FIX, and in (d,g) with sheep anti-human FX. Blots were imaged at 20 seconds (b-d) and 1 second (e-g) exposure times.

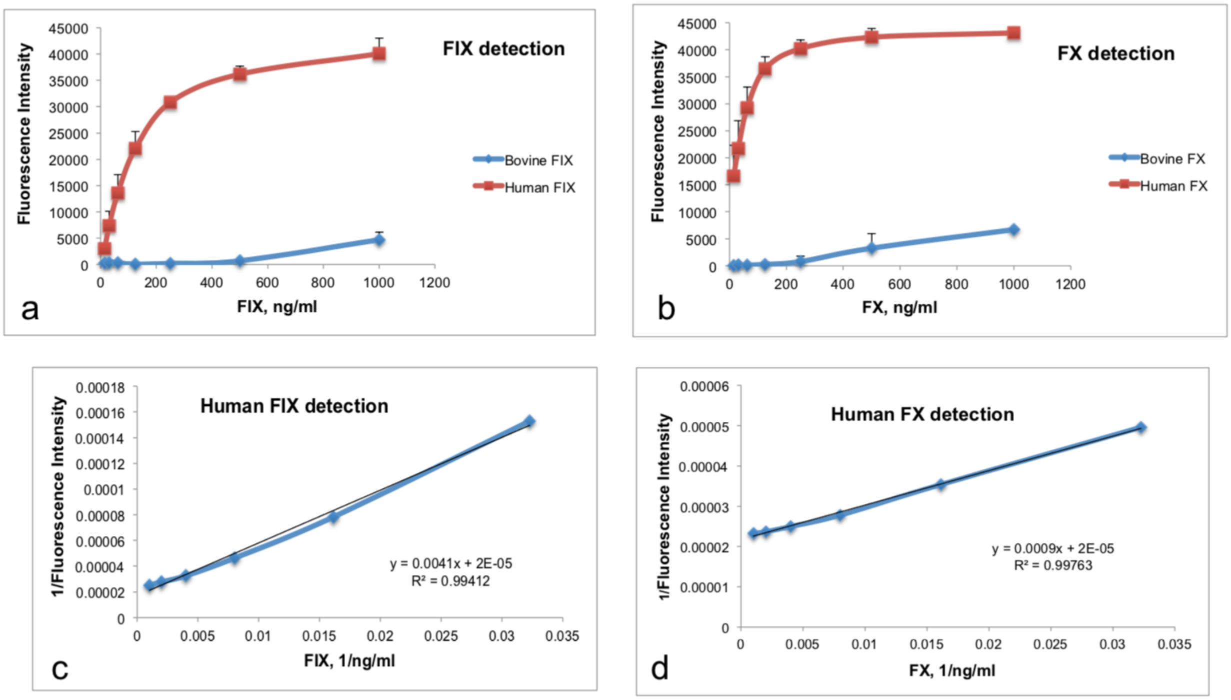

Fig. S2 Bovine factors are not detected with sheep antibodies made against human coagulation factors. (a) Detection of immobilized bovine FIX and human FIX using polyclonal sheep antibody against human FIX, followed by secondary donkey anti-sheep-HRP and a fluorescent HRP substrate. (b) Detection of immobilized bovine FX and human FX using polyclonal sheep antibody against human FX, followed by secondary donkey anti-sheep-HRP and a fluorescent HRP substrate. (c) Graph showing linear detection of human FIX protein using the sheep anti-human FIX antibody. (d) Graph showing linear detection of human FX using the sheep anti-human FX antibody. Results shown are means of triplicate measurements from 3 separate assays for each bovine and human protein plus SD.

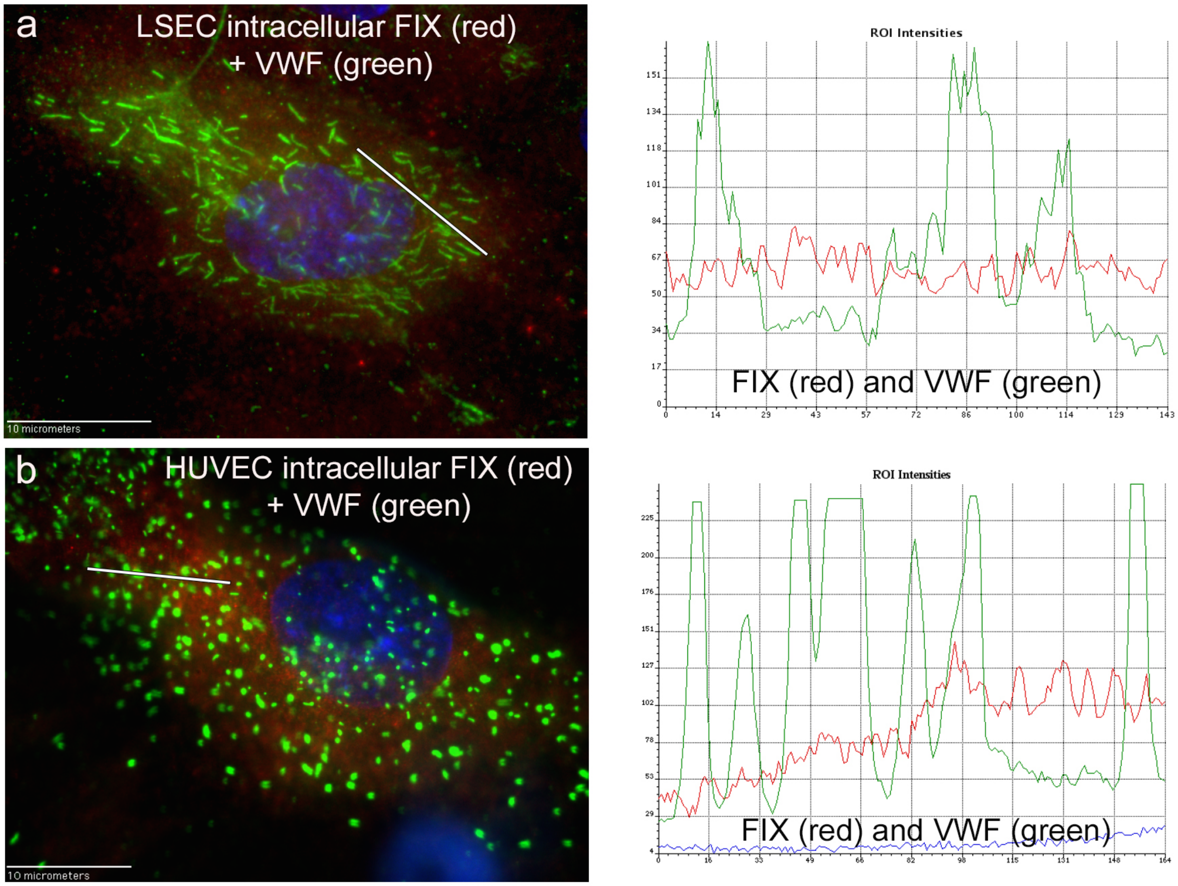

Fig S3. Images and graphs show the non-overlapping detection of intracellular FIX and VWF in LSECs and HUVECs. ECs were fluorescently labeled with antibodies against human FIX (sheep anti-FIX + donkey anti-sheep AF-647, red) and VWF in WPBs (rabbit anti-VWF + chicken anti-rabbit AF-488, green). Intensities (y-axis) from the red and green channels were measured along the white line that traverses through the EC WPBs and were plotted against the line length in pixels (x-axis). (a) LSECs, line length = 8 μm and (b) HUVECs, line length = 9.5 μm. ECs were imaged at 100X and the nuclei detected with DAPI. Selected images are representative of 15 images detecting both proteins. The region of interest (ROI) is the line where the intensities were measured.

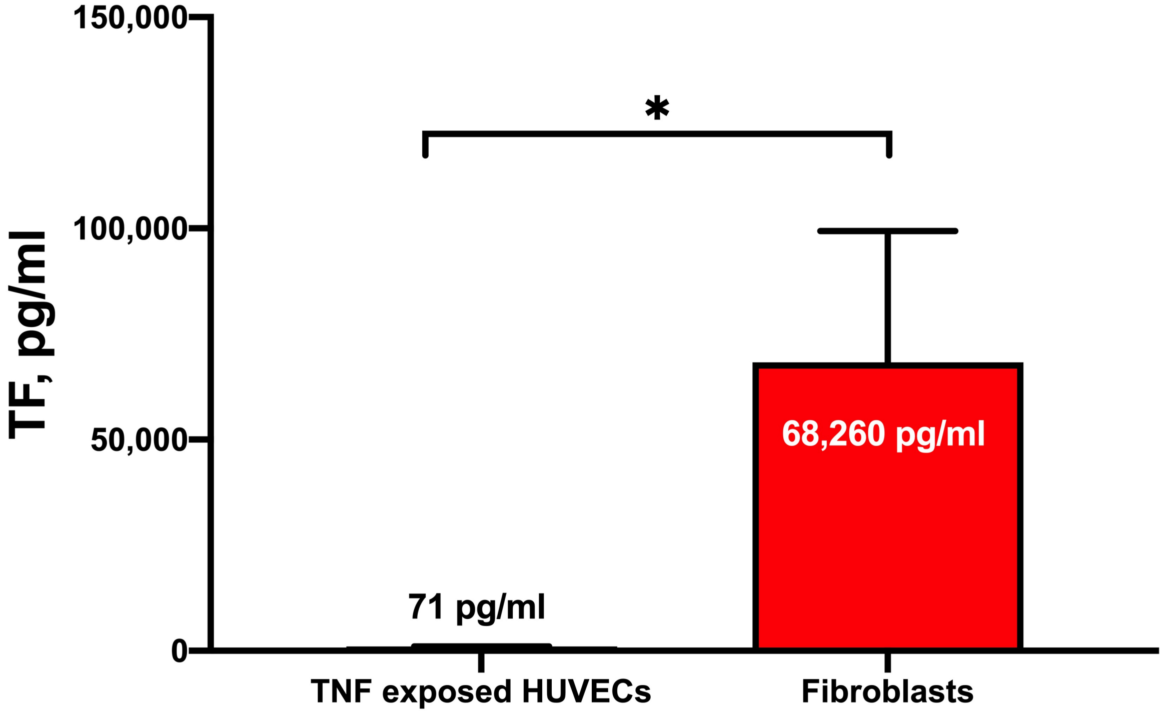

Fig. S4 Quantification of TF in TNF exposed HUVECs and untreated fibroblasts. Cell lysates for quantification of TF by ELISA were prepared from HUVECs exposed to 10 ng/ml TNF for 24 hours, and untreated fibroblasts. Concentrations of the proteins (means + SD) for TNF exposed HUVECs (n = 4), and fibroblasts (n = 5) were normalized to total lysate protein to account for cell number difference. *p<0.0001.

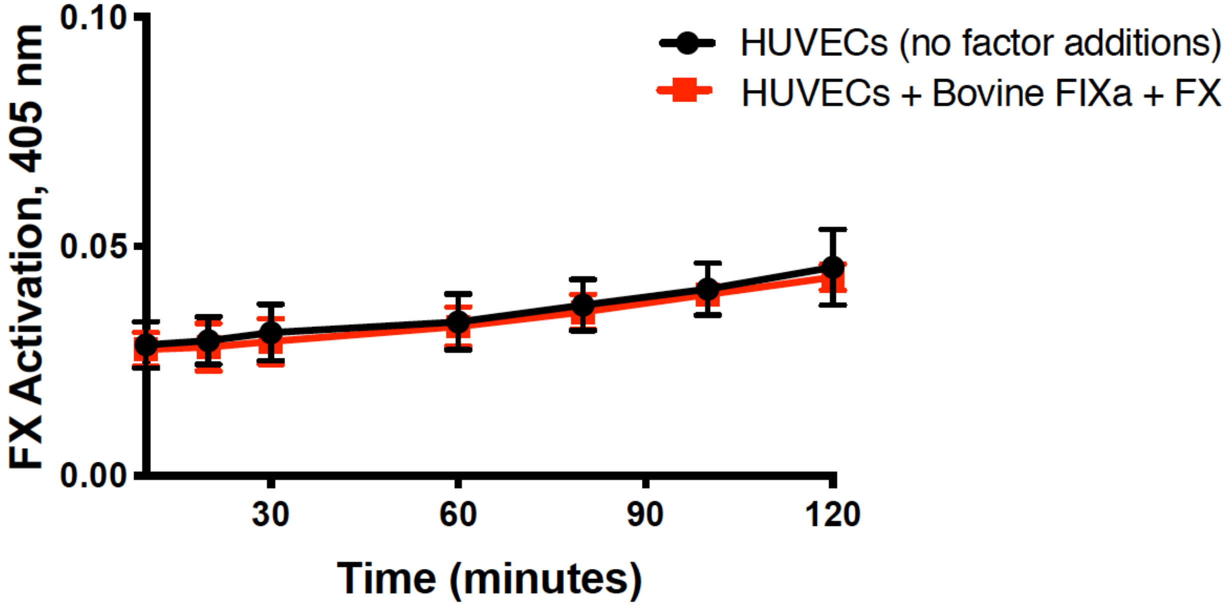

Fig. S5 FX activation on HUVEC surfaces following the addition of bovine activated FIX and bovine zymogen FX. FX activation was measured over time on HUVEC surfaces with (n = 8) and without (n = 8) the addition of 90 mIU of bovine coagulation factors, activated FIX (FIXa) and zymogen FX, immediately prior to the addition of the chromogenic substrate, S-2765. The measured FX activation was unchanged with the addition of bovine activated FIX and zymogen FX.

Coagulation Entrez gene ID and expression assay numbers

Gene	Entrez Gene	Assay ID #	Amplicon length	Gene Efficiencies (E)
THBD[*]	7056	Hs00264920_s1	91	1.90
PROCR	10544	Hs00197387_m1	70	1.96
TFPI	7035	Hs00409207_m1	111	1.87
GAPDH	2597	Hs99999905_m1	122	2.10
F3	2152	Hs0176029_m1	85	1.86
F7	2155	Hs01551992_m1	137	-
F9	2158	Hs01592597_m1	78	2[^]
F10	2159	Hs00984443_m1	154	1.78

Table S1 Coagulation Entrez gene ID and expression assay numbers. Amplified cDNA products were detected using TaqMan Gene Expression assays using 6-carboxyfluorescein-labeled probes that span target exon junctions. All information provided by the manufacturer can be found by searching these ID numbers. [*]The exception is *THBD* that does not have introns.[1] [^]Due to the low expression level of *F9,* serial dilutions were not possible to calculate the efficiency.

Supplemental Materials and Methods

Confirmation of antibody specificity

Western immunoblots

Gel lanes of coagulation protein standards contain 1.5 µg of FVII (HCVII-0030, 1.4 mg/ml); FIX (HCIX-0040, 4.4 mg/ml); and FX (HCX-0050, 7.7 mg/ml) (Haematologic Technologies). Other lanes include GMVEC lysate (2 µg protein), and StrepTactin-labeled molecular weight protein standards (WesternC, BioRad). Denatured, non-reduced samples in sodium dodecyl sulfate (SDS) were electrophoresed into 4-15% polyacrylamide gels (BioRad), stained with Bio-Safe Coomassie G-250 and transferred to PVDF membranes. After the gel was transferred to the membrane, the membrane was cut and detected with one of the three primary polyclonal sheep antibodies to each human coagulation protein separately; (FVII, PFVII-S, 9.1 mg/ml, ThermoFisher; FIX, PAHFIX-SAP, 3.1 mg/ml and FX, PAHFX-S, 10.6 mg/ml, both from Haematologic Technologies) diluted 1:8,000 in 1% bovine serum albumin (BSA) in PBS (BSA/PBS), followed by secondary donkey anti-sheep IgG-HRP plus StrepTactin-HRP conjugate. Blots were detected by chemiluminescent (Clarity Western ECL, BioRad) and digital imaging (ChemiDoc XRS, BioRad) with 1 and 20 second exposures.

Endothelial cell lysates for Western immunoblots

EC lysates for Western blots were prepared using P6 GMVECs grown in a T-75 flask. ECs were removed with 5 mM EDTA and pelleted cells were washed with cold PBS and re-centrifuged. Cell pellet was re-suspended in 100µl CelLytic M plus 0.5µl of protease/phosphatase inhibitor for 15 minutes on ice with rocking. Cell lysates were

centrifuged at 12,000g for 15 min at 4°C and aliquots of lysate supernatant with 6 mg/ml protein were stored at -80°C.

Specificity of polyclonal sheep anti-human FIX for human FIX and not for bovine FIX

Human FIX protein (HCIX-004, 4.4 mg/ml) and bovine FIX protein (BCIX-1040, 8.2 mg/ml) were diluted in 100 mM bicarbonate buffer, pH 9.6 and immobilized onto black 96-well plates at concentrations 1000–15 ng/ml overnight at 4°C. Proteins were washed with Tris-buffered-saline-Triton-X (TBS-T) and then blocked with 1% immunoglobin-free BSA in PBS (BSA/PBS). The primary detection polyclonal sheep antibody directed against human FIX (PAHFIX-SAP, 5.6 mg/ml) was diluted in BSA/PBS and incubated with sample (200 ng/well) for 1 hour at 37°C. Following TBS-T washing, wells were incubated with secondary donkey anti-sheep-HRP for 30 min. Fluorescence was measured in a plate reader (Tecan), after the addition of fluorescent substrate ADHP (10-Acetyl-3, 7-dihydroxyphenoxazine; AnaSpec,) with excitation at 530 nm and emission at 590 nm.

Specificity of polyclonal sheep anti-human FX for human FX and not for bovine FX

Human FX protein (HCX-0050, 7.7 mg/ml) and bovine FX protein (BCX-1050, 5.9 mg/ml) were diluted in bicarbonate buffer and immobilized onto black 96-well plates at concentrations 1000–15 ng/ml overnight as described for FIX protein. The rest of the protocol was followed except that the primary detection antibody was the polyclonal sheep antibody directed against human FX (PAHFX-S, 10.6 mg/ml) at 350 ng/well.

Measurement of FX activation on HUVEC surfaces with and without the addition of bovine activated FIX and bovine zymogen FX. FX activation on HUVEC surfaces was measured as described in the Methods section of the manuscript. In a subset of flasks, 100 µl of bovine activated FIX and zymogen FX (0.9 IU/ml) were added immediately prior to the addition of S-2765. The bovine activated FIX and FX solution is part of the Chromogenix Coatest SP4 Factor VIII kit (Diapharma), and contains 2.7 IU of lyophilized bovine factors with bovine albumin as a stabilizing agent. The factors were reconstituted with 3 mL of water prior to use. Duplicate samples were taken from the HUVEC containing flasks at timed intervals for a 120-minute time period and analyzed at 405 and 490 nm.

References

1 Jackman, R. W., Beeler, D. L., Fritze, L., Soff, G. & Rosenberg, R. D. Human thrombomodulin gene is intron depleted: nucleic acid sequences of the cDNA and gene predict protein structure and suggest sites of regulatory control. *Proceedings of the National Academy of Sciences of the United States of America* **84**, 6425-6429 (1987).
